# The Double-Edged Sword: Anterior Cruciate Ligament Reconstructions on Adolescent Patients—Growth Plate Surgical Challenges and Future Considerations

**DOI:** 10.3390/jcm13247522

**Published:** 2024-12-11

**Authors:** Alexandria Mallinos, Kerwyn Jones

**Affiliations:** 1 Rebecca D. Considine Research Institute, Akron Children’s Hospital, Akron, OH 44307, USA; 2 Department of Orthopedics, Akron Children’s Hospital, Akron, OH 44307, USA; kjones@akronchildrens.org

**Keywords:** ACL reconstruction, growth plate, physeal-sparing, transphyseal-sparing, review

## Abstract

The management of anterior cruciate ligament (ACL) injuries in pediatric patients presents unique challenges due to the presence of open growth plates in the proximal tibia and distal femur. Delaying ACL reconstruction until skeletal maturity may protect the physes but increases the risk of secondary injuries, such as meniscal tears and chondral damage, due to prolonged joint instability. Conversely, early surgical intervention restores knee stability but raises concerns about potential growth disturbances, including leg-length discrepancies and angular deformities. This narrative review examines current approaches to pediatric ACL management, highlighting the risks and benefits of both conservative and surgical treatments. Additionally, it explores the role of finite element modeling (FEM) as an innovative tool for pre-surgical planning. FEM offers a non-invasive method to optimize surgical techniques, minimize iatrogenic damage to growth plates, and improve patient outcomes. Despite its potential, FEM remains underutilized in clinical practice. This review underscores the need to integrate FEM into pediatric ACL care to enhance surgical precision, reduce complications, and improve long-term quality of life for young patients. By synthesizing available evidence, this review aims to provide clinicians with a comprehensive framework for decision-making and identify future directions for research in pediatric ACL reconstruction.

## 1. Introduction

The prevalence of anterior cruciate ligament (ACL) tears within pediatric populations has increased significantly over the past two decades, with an annual increase of 2.3% per year [1]. This growing trend of ACL ruptures in adolescents is a result of many different factors such as the rise in popularity of participation in youth sports, extensive training regimes and practices, and competitive sport cultures [2,3,4]. Injuries of this nature predominately occur around high school age, with females and males peaking around ages 16 and 17 [1].

Compared to their male counterparts, pediatric ACL injury rates occur more often in females, especially between the ages of 6 and 16 [1,5]. The literature has shown that when competing in equivalent sports, female athletes are two to eight times more likely to injure their ACL than males in both high school and collegiate settings [6,7,8,9,10,11,12]. Many factors play a role in why females have a higher incidence of ACL ruptures such as larger Q angles, greater tibial laxity, smaller ACL size, changes in geometry to the intercondylar notch, and even deviations to ligament biomechanics due to fluctuating hormone cycles [13,14,15].

For the pediatric knee, the proximal tibial and distal femoral physes play a critical role in bone growth and development and are ultimately the most at risk during ACL surgical intervention. Significantly impacting leg length, the distal femoral physis contributes to approximately 10 mm of growth per year [16,17,18,19]. Thus, at the time of skeletal maturity, the distal femoral physis will have contributed 70% and 40% to the femoral length and overall length of the limb [16,17,18,19]. Closure of the distal femoral physis occurs on average between the ages of 16 and 18 for boys and ages 14 and 16 in girls [20,21]. Over the lifetime, the tibial physis will grow approximately 6 mm per year and contribute to an average of 55% of the length of the tibia and 25% of the entire length of the leg [22,23]. Closure of the tibial physis will typically be completed by ages 13–15 in girls and by ages 15–19 years in boys [23]. As a result, the physes play a critical role in ensuring that normal limb length and alignment are achieved.

The rise in still-growing patients experiencing ACL tears poses significant challenges to surgical planning and techniques. The treatment of skeletally immature patients with ACL injuries has been the topic of much discussion within the literature, particularly due to the presence of the growth plate [24,25,26,27]. Disturbances to the physes occur in 2.6–24% of ACL reconstructions in the pediatric population [28,29]. Fortunately, the occurrence of surgical errors causing iatrogenic damage to the physes during ACL reconstruction are rare [30]. Leg-length discrepancies are the most common post-surgical complication caused by growth plate disturbances, followed by valgus angular deformities [31]. A systematic review [32] determined that leg-length discrepancies greater than 10 mm and 20 mm only occurred in 2.1% and 0.5% of skeletally immature patients. Angular deformities had an incidence of 1.3%, with femoral valgus malalignments being the most common [32]. Not only do growth plate disturbances impact the biomechanics of the knee, but severe cases also require additional surgical interventions, thus reducing the patient’s quality of life and impacting their ability to return to sports.

Current surgical techniques used in ACL reconstructions of adults, such as the anatomical transphyseal method, do not translate well into still-developing patients as these techniques place pediatric patients with open physes at risk for iatrogenic damage to the growth plate. In recent years, current techniques have moved away from conservative managements that consist of delaying surgery until skeletal maturation is reached to various surgical intervention methods. Despite there being numerous treatment methods explored in the literature, a clear gap in the literature exists as no clear consensus has been obtained in establishing a standard of care in the ACL reconstruction of skeletally immature patients. Many of the methods remain controversial due to extensive methodological inconsistencies and weaknesses [25,33]. At this point in time, ACL reconstructive techniques established in the literature in treating patients with open physes requires a delicate balance between safety and efficacy.

Although physeal-sparing techniques have been designed to mitigate the risk of damaging the growth plate, these approaches present a double-edged sword. They minimize the potential for growth disturbances but may introduce other complications like compromised graft strength or stability, particularly in young athletes, thus highlighting significant gaps and challenges in long-term outcomes that need further investigation.

The aim of this narrative review is to provide a comprehensive overview of the management of pediatric ACL injuries in patients with open growth plates. Specifically, this review will (1) describe the various surgical techniques used in pediatric ACL reconstruction and their associated challenges and post-surgical complications; (2) examine non-operative conservative treatment approaches, including their indications, benefits, and limitations; and (3) explore how advancements in non-invasive technologies, such as finite element modeling, can improve surgical planning and optimize outcomes. By addressing both operative and non-operative strategies, this review will provide clinicians with a framework for managing ACL injuries in skeletally immature patients while identifying areas for future research and innovation.

## 2. Methodology

This narrative review was conducted by performing a comprehensive literature search to ensure thorough coverage of the topic. Searches were conducted across databases including PubMed and Scopus using keywords such as “ACL reconstruction”, “physeal-sparing”, “skeletally immature”, “conservative”, “partial transphyseal-sparing”, and “transphyseal”. Articles were included if they focused on anterior cruciate ligament reconstruction in pediatric populations, were published within the last 25 years, and were available in English. The exclusions criteria included studies on adult populations and non-peer-reviewed articles. The selection process aimed to capture a broad spectrum of surgical techniques, outcomes, and biomechanical advancements relevant to the topic.

## 3. Delayed Non-Operative Conservative Treatment

Delaying ACL reconstruction until skeletal maturity is reached is a conservative approach to help preserve and protect the growth plates of pediatric patients [25]. Disrupting and crossing the physes during ACL surgery can lead to irreversible complications such as angulation deformities and leg-length discrepancies [34]. Delaying surgical interventions until the growth plates close helps minimize these risks. This non-operative treatment plan usually consists of activity restriction (such as the avoidance of pivoting and cutting activities and performing low-impact exercises such as swimming or stationary biking) and physical therapy combined with wearing an orthosis to help stabilize the knee.

Evidence supports exercise-based preoperative rehabilitation as an effective strategy to reduce the negative effects of delaying ACL reconstruction [35,36]. Programs targeting quadriceps and hamstring strengthening, proprioception, and knee stability help mitigate instability and decrease secondary injury risk during this period [35,36]. Patients’ categorization into copers, who maintain stability without surgery, and noncopers, who experience frequent instability, is essential. Copers typically demonstrate better neuromuscular control and fewer episodes of instability, making them better candidates for delayed reconstruction [36]. In contrast, however, noncopers are at higher risk for additional tissue injury and may benefit from earlier surgical intervention [36].

Thus, although good in theory, delaying ACL reconstruction until skeletal maturity has been associated with increased risk of additional damage to the knee joint [25]. Waiting for surgical intervention exposes the patient to a prolonged period of instability [25]. It has been well documented in the literature that pediatric patients who have been treated non-operatively for ACL reconstruction experience higher rates of secondary meniscal tears and chondral damage due to the increased instability of the knee joint [25,37,38,39,40,41,42,43,44,45]. This suggests that delayed operative treatment can have irreversible damage to the intra-articular soft tissue of the knee, especially if the patient has been classified as a noncoper [36]. Furthermore, for individuals who continue participating in pivoting or high-impact activities despite knee instability, the risk of these secondary injuries increases significantly [46]. This highlights the need for careful patient counseling on activity modification.

A meta-analysis showed that pathologic knee laxity was present in 75% of pediatric patients treated non-operatively as opposed to only 14% of laxity reported in those who underwent reconstruction surgery [47,48]. Notably, 25% of patient’s treated non-operatively maintained stable knee function without surgery [47,48], highlighting the variability in patient outcomes and the potential for successful conservative management in select cases. This difference can partially be explained by the distinction between copers and noncopers [35,36]. In addition, postponed surgical treatment of ACL injuries in the skeletally immature has been shown to have poor outcomes in post-operative return to sports in young athletic patients [37,49]. Functional instability of the joint makes it difficult for this population to return to pre-injury level activity and further increases the chances of reinjury or developing secondary complications [47].

At the time of writing, only two articles have been supportive of postponing ACL reconstruction until skeletal maturity is reached [50,51], despite much of the literature warning against this method of treatment. When comparing the outcomes of 13 skeletally immature patients whose surgeries were delayed by at least 16 months to 116 patients who received reconstructive surgery approximately 14 weeks after injury, Wood et al. [50] found no significant differences between the two groups when it came to functional outcomes and the development of secondary injuries. The authors argue that postponing surgical intervention until skeletal maturity is reached can be achieved safely with minimal post-operative complications if the patient restricts all activity and wears an orthosis during this non-operative period [50]. As discussed by Al-Hadithy et al. [25], there are many limitations to this study such as extreme differences in group sizes (13 vs. 116) and its lack of clarity in describing the rehabilitation programs utilized by the patients [25,50]. In addition, the small sample size of the delayed operative group (n = 13) limits the generalizability and statistical power of the findings.

More recently, Ekås et al. [51] performed an 8-year follow-up on pediatric patients (less than 13 years of age at the time of ACL rupture) who received non-operative treatment plans. Although reporting that 45% of the patients responded well to conservative treatment (not requiring surgical intervention), 55% of patients had to eventually undergo ACL reconstruction, with 36% requiring meniscectomies [51]. The patients with better conservative treatment outcomes were classified as copers [51]. The authors viewed this conservative technique favorably due to its comparable performance in functional outcomes such as muscle strength and hop performance against those who had surgical intervention shortly after the onset of injury [51]. Despite comparable functional performances, the authors failed to consider the negative consequences of delayed ACL reconstruction since the majority of patients eventually underwent ACL reconstruction in addition to one in three patients developing secondary meniscal damage due to prolonged joint instability. It would be a disservice to patients to not consider the long-term repercussions that delaying surgery can have, especially if they can lead to secondary injuries or irreversible cartilage damage, which was not a post-surgical outcome examined by the authors.

Conservative, non-operative treatment plans tend to be more successful in skeletally immature patients who have a partial tear to the ACL [52,53,54,55,56]. Indications of non-operative treatment include skeletally immature patients who have a negative pivot shift test, have considerable growth left, and ruptured less than 50% of the ligamentous fibers [52,55,56,57]. Additional considerations like muscle strength imbalances and performance in functional tests such as single-legged hop test and isokinetic muscle strength measures, are essential for evaluating joint stability and neuromuscular control [58,59]. These papers emphasize that conservative treatment or surgical intervention for partial ACL tears in skeletally immature patients can vary depending on factors like growth potential, knee stability, and the severity of the injury.

In summary, the decision to delay ACL reconstruction until skeletal maturity must balance the risks of growth disturbances against the potential for secondary iatrogenic injuries. While this treatment method protects the still-developing growth plates, it also increases the risk of further joint damage. Therefore, the decision to implement a delayed non-operative treatment plan in skeletally immature patients should be made on a case-by-case basis.

## 4. Surgical Interventions

Numerous operative ACL reconstruction techniques have been developed in order to help restore the stability of the knee but at the risk of surgically induced damage to the still developing physes. Intra- or extra-articular techniques utilize a variety of methods that are either partial transphyseal, physeal-sparing, or transphyseal in nature. This section will explore these different operating methods and discuss their advantages and disadvantages.

### 4.1. Physeal-Sparing Reconstruction

Utilizing both extra- and intra-articular methods, physeal-sparing surgical interventions aim to avoid crossing into both the tibial and femoral physes. Popular techniques will utilize over-the-top [52,60] or all-epiphyseal [61,62,63] techniques to spare injury to the still-developing physes. Because the physeal-sparing method avoids crossing the growth plate in skeletally immature patients, these methods are recommended in prepubescent patients of younger skeletal age (Tanner stages 1–2) [24]. Compared to partial transphyseal-sparing and transphyseal approaches, physeal-sparing techniques have reported better post-operative restoration of knee laxity but have comparable clinical outcomes to the other surgical techniques [64].

Over-the-top reconstruction methods position the graft “over the top” of the lateral femoral side and are fixated to the tibial metaphysis [25]. In contrast, the all-epiphyseal technique aims to achieve a more anatomically correct graft position which drills into the epiphyses of the tibia and femur while avoiding damage to the growth plates [65]. The Micheli reconstruction technique integrates both intra- and extra-articular methods in which an iliotibial band is passed through the lateral femoral condyle and fixed over the anterior tibial [25]. Positive patient outcomes with no growth disturbances have been reported in the literature when utilizing physeal-sparing techniques such as over-the-top [60,66], all-epiphyseal [61,62,63], and the Micheli ACL reconstruction method [6,67,68].

When reviewing the surgical outcomes of physeal-sparing ACL reconstructions, Wong et al. [65] determined that both all-epiphyseal and over-the-top techniques are successful, producing favorable International Knee Documentation Committee (IKDC) grades (between 88.5 [69] and 100 [70]) and Lysholm scores (between 93 [69] and 97.9 [71]). IKDC and Lysholm scores are outcome measures that evaluate knee function, sports activity, and symptoms, where higher scores indicate fewer symptoms and better overall knee function. Although the prevalence of growth plate disturbances occurring with this method were rare, over-the-top reconstructions had a higher incidence of angular deformities while all-epiphyseal patients had a higher occurrence of physeal overgrowth [65]. All-epiphyseal reconstructions had a higher re-rupture rate (9%) compared to over-the-top surgeries (7.2%), of which 82% required surgical revision [65].

Despite the preventative measures taken in minimizing damage to the physes, iatrogenic injury to the growth plates was still shown to occur in numerous physeal-sparing surgical methods [31,65,69,72,73,74]. A systematic review has shown that transphyseal and physeal-sparing ACL reconstructions have similar incidence rates of growth plate disturbances resulting in angular deformities and leg-length discrepancies [74].

Shockingly, Collins et al. [31] performed a systematic review identifying all cases of growth plate disturbances in skeletally immature patients after ACL reconstruction. They found that of patients who experienced leg-length discrepancies due to overgrowth, 50% of this demographic underwent physeal-sparing techniques [31]. This is consistent with findings in the meta-analysis performed by Wong et al. [72], where approximately 64% of patients who experienced a leg-length discrepancy with overgrowth underwent a physeal-sparing ACL reconstruction. Furthermore, physeal-sparing patients had a greater likelihood of developing angular deformities (33%) compared to partial transphyseal-sparing methods (11%).

These observations of growth disturbances are contrary to the popular rhetoric that deem physeal-sparing surgical methods safe for skeletally immature patients. This demonstrates growth disturbances can occur with any reconstruction method, thus highlighting the importance of proper surgical planning and technique.

### 4.2. Partial Transphseal-Sparing Reconstruction

Partial transphyseal-sparing reconstruction methods aim to balance the critical need for knee joint stability with the protection and integrity of the growth plate in skeletally immature patients. This technique provides a compromise between anatomical transphyseal and physeal-sparing procedures as it aims to maintain a more native ACL position while minimizing physeal drilling, ultimately decreasing the risk of growth disturbances and angular deformities.

During this surgical procedure, the graft placement is carefully mapped out to avoid crossing at least one of the physes. Typically, the graft will pass through a transphyseal tibial tunnel and then will be fixed to the lateral femoral metaphysis [25]. Techniques such as the all-epiphyseal or over-the-top methods are utilized so that the femoral portion of the graft is positioned away from the growth plate [24].

Since the femoral physis is spared with this surgical technique, it reduces the likelihood of growth plate disturbances to the femur, which contributes to the greatest percentage of the limb’s overall length [16,17,18,19]. On the tibial side, the transphyseal tunnel is drilled with the tunnel size and angle adjusted in a manner that minimizes the risk of growth disturbances. Minor drilling into the tibial physis is viewed more favorably since it closes at an earlier age and does not contribute as much to the overall length of the limb as the femoral physis does.

Many partial transphyseal reconstruction techniques utilize an all-epiphyseal femoral tunnel and drill a reasonably vertical tibial tunnel [24,63,75,76,77]. In an MRI-modeling based study, Kercher et al. [78] reported that more vertical drilling angles decrease the percentage of physeal volume removed with the graft diameter having the greatest impact on the volume of the physeal injury. In fact, many animal models have observed growth plate disturbances occurring, with as little as 7% of the physeal volume being removed [79,80]. Computer modeling techniques have shown that increasing the drill diameter from 6 to 9 mm more than doubles the percentage of physis volume lost for both the tibial and femoral physis [81]. Fortunately, less than 3% of physeal volume is removed when drilling an 8 mm tunnel through the physis [78] with many studies reporting favorable patient outcomes in utilizing this surgical technique [63,75,76,82,83,84,85].

Regardless of the good functional outcomes, drilling into the unclosed physis comes with risks. In particular, Chambers et al. [85] reported the incidence of growth disturbances in skeletally immature patients receiving partial transphyseal ACL reconstruction. Although they found that 16.7% of their patient group experienced postoperative growth plate disturbances, there was a staggering incidence of growth disturbance occurring in 66.7% of patients who had more than 5 years of growth remaining [85]. This demonstrates that although this technique can produce successful outcomes with a low risk of growth plate disturbance, patient-specific factors such as skeletal age should be taken into consideration during surgical planning.

Due to the risk of physeal damage and ultimately disturbances with bone development and angle deformities, this method is not recommended for younger patients with significant growth remaining. Partial transphyseal reconstruction techniques are typically reserved for adolescents presenting at Tanner stage 2–3 [24], to help minimize the impact of growth disturbance if physis damage were to occur.

### 4.3. Transpyhseal Reconstruction

Transphyseal ACL reconstructions consist of drilling tunnels into both femoral and tibial physes with the aim of positioning the graft in a more anatomically correct, native position [26]. This approach mirrors the reconstruction techniques used in adult populations in order to achieve better biomechanical alignment and provide stronger graft fixation. This technique requires careful surgical planning to avoid crossing the physes and comes at a greater risk for post-operative growth disturbances or angulation deformities [25].

When it comes to transphyseal ACL reconstruction on skeletally immature patients, strategic locations and orientations of the tunnel, in combination with drilling diameter, have been shown to be especially advantageous [86]. In combination with a more vertical drilling angle [78], tibial tunnels positioned with a more distal and medial orientation result in a smaller percentage of tibial physeal volume lost [87]. Greater femoral physis damage occurs when drilling passes through the anteromedial portal compared to those using the trans-tibial technique [86,88,89]. However, physis damage can be minimized if the anteromedial portal is drilled perpendicular to the physis at a more vertical orientation [86,90,91]. Due to a greater oblique angle, outside-in transphyseal tunnels produce greater physis volume loss than the trans-tibial technique [92]. In addition, other transphyseal surgical techniques that help avoid growth disturbances include sparing the tibial tuberosity to prevent angular deformities, utilizing soft tissue grafts, and avoiding thermal damage to the physes [86,93].

Several studies have reported that this technique is safe to perform in adolescent (Tanner stage 2–3) [94,95,96] and prepubescent (Tanner stage 1–2) [90,91,97] populations. Although the transphyseal ACL reconstruction technique has been shown to have a positive impact on clinical outcomes in skeletally immature patients [98], roughly 5% of these patients will experience growth plates disturbances [97,99,100]. Wong et al. [72] conducted a meta-analysis on post-surgical complications in pediatric ACL reconstructions. Of the three surgical techniques (physeal sparing, transphyseal, and partial transphyseal sparing), transphyseal ACL reconstruction patients had a higher incidence of developing angular deformities and leg-length discrepancies of at least 1 cm. Thus, due to the higher likelihood of iatrogenic physis damage, it is recommended to perform this surgical technique on adolescents at Tanner stage 2 or later, who are nearing the end of their growth (<1 cm) and would not be as greatly impacted from growth disturbances [24].

## 5. Discussion

### 5.1. Clinical Considerations

Numerous treatment methods have been developed in order to perform ACL reconstructions on skeletally immature patients with the goal of minimizing the risk of iatrogenic damage to the still-developing growth plate (Table 1). Despite the various surgical techniques, a clear gap in the literature exists as no clear current standard of care has been established to develop a surgical plan for ACL reconstruction in skeletally immature patients. All of the surgical methodologies explored in the previous section place the patient at risk for experiencing growth plate disturbances, even the physeal-sparing techniques. As a result, these approaches present a double-edged sword. Although patients benefit from restoring joint stability through surgical intervention, which minimizes the risk of the patient developing secondary cartilage damage and meniscal tears [25,34,37,38,39,40,41,42,43,44,45], the current reconstructive techniques have the potential to cause growth disturbances, which can result in leg-length discrepancies and angular deformities [31,32,34,74]. Ultimately, we can restore joint stability but at the cost of potentially risking growth disturbances.

Placing the reconstructed graft close to the anatomical position of the native anteromedial bundle is believed to be beneficial for reapproximating the isometric position of the anteromedial bundle of the ACL, thus leading to overall improved stability of the graft. The goal of transphyseal ACL reconstructions and all-epiphyseal ACL reconstructions is to reapproximate this relationship, whereas the physeal-sparing reconstructions and partial transphyeal-sparing reconstructions cannot reproduce this native anatomy. Despite this non-anatomic positioning, biomechanical analyses of these two techniques demonstrate overall very good postoperative stability in cadaveric models.

An in vitro analysis in 10 cadaveric knees revealed that all-epiphyseal ACL reconstruction shifted contact anteriorly on the tibia, reducing the change caused by ACL injury [101]. With the quadriceps and hamstrings loaded, ACL reconstruction shifted contact anteriorly by approximately 3 and 2 mm on the medial and lateral plateaus, respectively [101]. In a cadaveric study performed by Kennedy et al. [102] on six knees, all-ephiphyseal, transtibial over-the-top, and iliotibial band ACL reconstructions were evaluated. All three techniques improved the overall stability of the knee compared to the ACL-deficient knee. However, the iliotibial band reconstruction yielded the best overall rotational and anterior–posterior translational control. Interestingly, it also yielded some degree of over-constraint to rotational control at some flexion angles. This may indicate that, although the graft is not fixed near the anatomic footprint of the anteromedial bundle on the femur and tibia, excellent stability is still imparted in both the anterior–posterior and rotational planes using this technique.

To our knowledge, little is known about the potential for growth of the ACL graft as a child grows following placement. As the child grows, does the graft grow with them or does it stay at the same length over time, thus leading to the potential for over-constraining of the joint or possible rupture due to over-tightening? At this point in time, follow-up MRI studies on 10 immature patients that had ACL reconstructions demonstrated a 25% decrease in hamstring graft diameter on MRI one year following surgery [103]. However, more efforts need to be made in order to address the gaps in the literature pertaining to how ACL geometry changes throughout growth and development.

All-epiphyseal ACL reconstructions and iliotibial band reconstructions performed by the Kocher technique yield similar re-rupture rates of 9.0% and 7.0%, respectively [60]. Although the risk of growth arrest is very low for both techniques, the all-epiphyseal technique has a higher chance of growth arrest (seven patients) than the over-the-top of the femoral group position (one patient). Most surprisingly, none of these patients had undergrowth as all experienced overgrowth. Likewise, there were a total of four angular deformities, all in the over-the-top femoral group [65,104]. Cruz et al. [104] demonstrated that overall, the all-epiphyseal procedure, although technically challenging, is safe, with an overall complication rate of 16.7% and the most common cause of complication being re-rupture rate of 10.7%. Only one patient had a leg-length discrepancy at 1 cm.

Despite the extensive literature devoted to examining the effectiveness of the various pediatric ACL reconstruction techniques, there still remains no clear standard of care for ACL reconstruction in skeletally immature patients. Each technique explored in this review comes with risks for iatrogenic damage to the physes, which highlights the need for the importance of proper surgical planning and techniques. Thus, significant efforts must be made in order to improve surgical methodologies that not only are consistent across patients but also minimize post-operative risks and improve patient’s overall quality of life.

### 5.2. Future Directions: Finite Element Analysis

Finite element modeling (FEM) is a computational technique used to simulate and analyze the behavior of structures under various physical conditions. In the context of orthopedic research, FEM is often applied to study the biomechanics of joints, ligaments, and bones. By dividing the complex structure into smaller, finite elements, the model can evaluate stresses, strains, and displacements with high precision [105,106,107,108,109,110]. This approach is particularly valuable for understanding the mechanical implications of ACL injuries and the effects of different treatment strategies on growth plates in skeletally immature patients. FEM provides critical insight into surgical planning and the development of personalized treatment approaches.

Back in 2011 and 2018, Kennedy et al. [102] and Richter et al. [26] discussed how advancements in technology can be utilized to improve surgical techniques and planning when it comes to ACL reconstruction in skeletally immature patients. Within the last decade, significant improvements have been made to finite element modeling techniques such that they provide the opportunity to explore the patient-specific knee biomechanics in an accurate and novel manner [105,106,107,108,109,110,111,112]. The integration of this technology into surgical planning is especially advantageous when working with vulnerable populations such as pediatrics because it offers a way to explore the in vivo biomechanical environment through non-invasive means.

FEM holds immense potential in addressing challenges associated with ACL reconstruction in skeletally immature patients. One notable application of FEM that would be extremely valuable is its ability to evaluate the effects of various drilling techniques on physeal integrity. For example, FEM can simulate different tunnel angles and assess the extent of growth plate damage, allowing surgeons to identify the most biomechanically sound and minimally invasive approach. By providing precise data on stress distributions and deformation patterns, FEM can guide surgeons in optimizing surgical techniques to preserve growth potential while ensuring graft stability. Furthermore, FEM enables the study of different graft types and orientations, helping to evaluate their biomechanical compatibility with the pediatric knee joint, which is crucial for maintaining stability and preventing complications.

Another critical application of FEM is its use for patient-specific surgical planning. By incorporating individual anatomical and biomechanical data, FEM can simulate potential outcomes for various surgical scenarios. This capability is particularly beneficial for pediatric patients with unique growth patterns or complex injuries. For instance, FEM can model the impact of surgery on future growth trajectories and joint alignment, providing surgeons with a comprehensive tool to anticipate and mitigate potential complications, such as leg-length discrepancies or angular deformities. This individualized approach aligns with the growing emphasis on precision medicine and has the potential to significantly enhance patient outcomes.

Despite its promise, integrating FEM into clinical practice comes with challenges. Developing accurate FEM models requires high-resolution imaging, such as MRI or CT scans, and sophisticated computational resources. These requirements can limit its accessibility in clinical settings. Additionally, FEM simulations are highly dependent on the quality and accuracy of input data, such as material properties of pediatric bone and cartilage, which may vary significantly between individuals. Variability in these parameters can affect the reliability of FEM predictions. Finally, the lack of standardized protocols for FEM application in pediatric ACL reconstruction further hinders its adoption. Addressing these challenges through collaborative research and development is essential for realizing the full potential of FEM in improving surgical outcomes and patient quality of life.

This area still remains deeply understudied in pediatric applications. A simple PubMed search of “ACL, biomechanics, pediatric, finite element” produces only six articles, one of which is the article written by Richter et al. [26]. At the time of publication, only one article has integrated finite element methods into improving surgical drilling techniques used in ACL reconstruction in skeletally immature patients [88]. Kachmar et al. [88] used a finite element analysis to determine the percent of physeal volume removed and the location of the growth plate disturbances during anteromedial and transtibial reconstruction.

Despite the promising results, finite element modeling still remains a severely underutilized tool in optimizing ACL reconstructions in patients at risk of iatrogenic growth plate disturbances. Various surgical techniques can be examined through finite element exploration such as drilling angle, graft orientation and type, patient-specific outcomes, the changes imposed during growth and development, and even the extent of physeal volume damage. Finite element modeling also offers the opportunity to provide pre-surgical visualization and perform patient-specific reconstruction analyses to aid in surgical planning optimization with minimal physis damage. As a result, significant contributions must be made to this severely understudied field in order to improve surgical outcomes with minimal risk to still-growing patients.

### 5.3. Limitations

This review is not without its limitations. Firstly, as a narrative rather than a systematic review, the selection of studies may introduce bias, and some relevant evidence may have been missed. Additionally, the lack of standardized methodology for the literature review limits reproducibility. While efforts were made to include comprehensive and representative studies, the scope of the review was restricted to articles available in English, which may exclude valuable findings published in other languages. Furthermore, variations in study designs and patient populations across the included literature may reduce the generalizability of the conclusions.

Despite these limitations, this narrative review provides valuable insights into the management of ACL injuries in skeletally immature patients. By synthesizing existing evidence, it highlights key considerations for conservative and surgical treatment approaches, identifies gaps in the literature, and offers guidance for clinicians in tailoring patient-specific management strategies. This work serves as a foundation for future research and systematic reviews in this area.

## 6. Conclusions

Despite the increase in pediatric ACL injuries, a significant gap in the literature exists such that no clear consensus has been obtained in establishing a standard of care in the ACL reconstruction of skeletally immature patients. Growth disturbances such as leg-length discrepancies and angular deformities can occur in any of the three main surgical methods explored in this manuscript. With no clear “gold standard” reconstruction technique established; proficient surgical technique is vital to ensure minimal damage to the physis. Finite element methodologies make it possible to explore surgical planning in a non-invasive and novel manner. Although finite element technology can significantly improve ACL reconstruction on skeletally immature patients through pre-surgical visualization and optimization of surgical outcomes, it still remains a severely underutilized tool. Significant effort should be made to integrate finite element analyses into pediatric ACL reconstruction care to not only minimize the risk of iatrogenic damage to the growth plates but also improve surgical outcomes and patient quality of life.

## Figures and Tables

**Table 1:** Summary of the pros and cons of pediatric ACL management approaches.

Approach	Pros	Cons
Delayed Non-Operative Conservative Treatment	Avoids surgical risks entirely. Allows for potential growth plate preservation.	Higher risk of secondary injuries such as chondral damage and meniscal tears. May not adequately address joint instability in active patients.
Physeal-Sparing Reconstruction	Avoids drilling through the growth plates. Minimizes the risk of growth disturbances.	Technically challenging. Limited to younger patients with significant growth remaining.
Partial Transphyseal-Sparing Reconstruction	Balances anatomical reconstruction with physeal preservation.	Requires careful planning to avoid significant physeal damage. Increased surgical complexity compared to physeal-sparing techniques.
Transphyseal Reconstruction	Anatomical reconstruction. Stronger graft fixation. Suitable for older children nearing skeletal maturity.	Higher risk of growth plate injury. Potential for growth disturbances, including leg-length discrepancies and angular deformities.

## Data Availability

Not applicable.
